# Safety of phosphatidylserine containing omega-3 fatty acids in non-demented elderly: a double-blind placebo-controlled trial followed by an open-label extension

**DOI:** 10.1186/1471-2377-11-79

**Published:** 2011-06-28

**Authors:** Veronika Vakhapova, Yael Richter, Tzafra Cohen, Yael Herzog, Amos D Korczyn

**Affiliations:** 1 Neurology Department, Tel-Aviv Sourasky Medical Center, Tel-Aviv, Israel; 2 Enzymotec Ltd. P.O.Box 6, 23106, Migdal HaEmeq, Israel; 3 Sieratzki Chair of Neurology, Tel-Aviv University Medical School, Ramat Aviv, Israel

## Abstract

**Background:**

Phosphatidylserine (PS) is a naturally occurring phospholipid present in the inner leaflet of mammalian plasma membranes. Administration of PS extracted from bovine cortex (BC-PS), which contains high levels of omega-3 long chain polyunsaturated fatty acid (LC-PUFA) attached to its backbone, resulted in positive effects on brain functions such as learning and memory. Recently, a novel marine-sourced PS with omega-3 LC-PUFA attached to its backbone was developed (PS-DHA). In the present study, we evaluated the safety profile of the novel PS preparation in non-demented elderly with memory complaints. The efficacy study of this novel formulation indicated that PS-DHA may ameliorate cognitive deficits in non-demented elderly population.

**Methods:**

157 non-demented elderly participants with memory complaints were randomized to receive either PS-DHA (300 mg PS/day) or placebo for 15 weeks. Standard biochemical and hematological safety parameters, blood pressure and heart rate were evaluated at baseline and endpoint. 122 participants continued into an open-label extension for additional 15 weeks, in which they all consumed PS-DHA (100 mg PS/day) and were evaluated for their blood pressure, heart rate and weight at endpoint. Adverse events were monitored throughout the double-blind and open-label phases.

**Results:**

131 participants completed the double-blind phase. No significant differences were found in any of the tested safety parameters between the study groups, or within each group. 121 participants completed the open-label phase. At the end of this phase, there was a reduction in resting diastolic blood pressure and a slight weight gain among participants who consumed PS-DHA for 30 weeks.

**Conclusions:**

The results of this study indicate that consumption of PS-DHA at a dosage of 300 mg PS/day for 15 weeks, or 100 mg PS/day for 30 weeks, is safe, well tolerated, and does not produce any negative effects in the tested parameters.

**Trial registration:**

clinicaltrials. gov, identifier: NCT00437983

## Background

Phosphatidylserine (PS) is a naturally occurring phospholipid present in the inner leaflet of mammalian plasma membranes. In humans, PS is most concentrated in the brain where it comprises 15% of the total phospholipid pool. PS has been shown to play a key role in the functioning of neuron membranes, such as signal transduction, secretory vesicle release and cell-to-cell communication [1].

The administration of PS extracted from bovine cortex (BC-PS) has positive effects on brain function. BC-PS was shown to improve learning and memory in age-associated memory impaired subjects [2], to enhance behavioral and cognitive parameters in geriatric patients [3], and to improve cognitive performance of Alzheimer's disease (AD) patients [4,5]. Although the primary objective of clinical studies involving BC-PS was to test efficacy, no significant adverse events were reported following oral administration of BC-PS at doses of 300-400 mg/day for up to 3 months [2-8]. In the largest double-blind, placebo-controlled trial [3], comprising 494 participants, only one subject dropped out because of an adverse event, as compared to seven drop-outs from the placebo group.

Due to safety concerns about potential contamination by bovine spongiform encephalopathy (BSE) prions in recent years, alternatives to BC-PS, such as soy derived PS (SB-PS), have been developed. SB-PS however, differs considerably in its fatty acid composition as compared to mammalian brain PS, and while SB-PS was shown to attenuate both physical [9] and mental stress [10,11], further research is required to determine its ability to promote cognitive functioning [12,13]. The safety of SB-PS was tested in a double-blind placebo controlled study on 120 elderly [14]. Hematological and biochemical parameters along with vital signs and adverse events were evaluated after 6 and 12 weeks of treatment. The study conclusion was that SB-PS is safe for administration to older persons if taken up to a dosage of 600 mg/day [14].

To gain the benefits of mammalian PS without the attendant risks, a safe, marine-sourced PS with omega-3 long chain polyunsaturated fatty acid (LC-PUFA) attached to its backbone was developed. This compound was recently found to improve the symptoms of children with impaired visual sustained attention [15] and to protect middle-aged rats from scopolamine-induced deleterious effects [16]. However, the biochemical and hematological tolerability of PS with omega-3 attached to its backbone have not been presented until now.

In the present report we describe the safety of a novel formulation of PS with omega-3 LC-PUFA, mainly docosahexaenoic acid (DHA), attached to its glycerol backbone (PS-DHA), in non-demented elderly. The relevant safety parameters were derived from biochemical and hematological variables, examination of vital signs and adverse events monitoring during the 15 weeks double-blind placebo controlled study and from examination of vital signs and adverse events monitoring during the 15 additional weeks of the open-label extension phase. The efficacy of this novel formulation has also been tested in this population and the results of the double-blind phase are reported elsewhere [17]. Briefly, the study results indicated that PS-DHA may improve cognitive performance in non-demented elderly with memory complaints as PS-DHA administration resulted in an improvement of verbal immediate memory and in higher responders rates in comparison with the placebo group [17].

## Methods

### Subjects

Participants were recruited through advertisements in senior citizens homes, hospitals, and newspapers. Approximately 700 elderly were screened for enrollment to the study. Out of them 157 non-demented participants with memory complaints met the previously described inclusion criteria [17]. Briefly, eligible participants were non-demented men or women between the ages of 50 and 90 years, with complaints of memory loss [18] and no evidence of a condition that could produce cognitive deterioration including AD, Parkinson's disease, stroke, normal pressure hydrocephalus, and other brain lesions including tumors, renal, respiratory, cardiac, and hepatic disease, diabetes mellitus, endocrine, metabolic or hematological disturbances unless well controlled, and malignancy not in remission for more than two years. Concomitant use of drugs or supplements affecting cognitive function was prohibited.

The study was conducted according to the principles of the Declaration of Helsinki and good clinical practice. The protocol was approved by the Ethics Committee of the Sourasky Medical Center, Tel-Aviv, Israel, and all volunteers gave written informed consent prior to participation.

### Study Design

The study was designed as a single-center, randomized, double-blind, placebo-controlled, 15 weeks study, followed by an open-label extension of additional 15 weeks. At the first, double--blind, phase, participants were randomized according to a computerized process based on 6 and 8 blocks, in a 1:1 ratio stratified by gender, to receive three capsules per day of PS-DHA or a matched identically looking placebo (cellulose). The daily PS-DHA dosage provided 300 mg PS and 79 mg DHA+EPA (DHA:EPA ratio of 3:1). During the second, open-label, phase, participants consumed one capsule a day of PS-DHA. The daily dosage provided 100 mg PS and 26 mg DHA+EPA. PS-DHA (Vayacog™) was supplied by Enzymotec, Migdal HaEmeq, Israel.

Safety was evaluated by clinical laboratory assessments including biochemical and hematological parameters at baseline and endpoint of the double-blind phase and by adverse events recording, physical examination and measurement of vital signs and weight at baseline, week 7 and endpoint (week 15) of the double-blind phase and at the end of the open-label extension (week 30).

Blood samples were analyzed by the American Medical Laboratories (AML), Herzliya, Israel. Adverse events were monitored and recorded at each visit and by telephone contact every other week.

### Laboratory Parameters

Biochemical parameters consisted of potassium, sodium, calcium, phosphorus, chloride, glucose, creatinine, blood urea nitrogen (BUN), bilirubin, total protein and lipid profile (total cholesterol, triglycerides, HDL, and LDL), alanine-aminotransferase (ALT), aspartateaminotransferase (AST), and alkaline phosphatase. Hematology consisted of red blood cell count, hematocrit, hemoglobin, white blood cell count and differential, platelets, MCV, MCH, and MCHC.

### Physical parameters

The parameters assessed were weight, resting systolic and diastolic blood pressure, and pulse rate.

### Statistical Analysis

Two-sided Student's t-test for dependent samples was used to analyze changes between different points in time in the tested parameters, in the whole group and in each gender separately, for both arms.

Two-sided t-test for independent samples was used to analyze differences between arms in the change between baseline and week 15 in blood parameters, vital signs and weight, in the whole group and in each gender separately and to detect any difference between groups in the frequency of adverse events.

Pearson's chi-square test for categorical variables was used to analyze the differences between groups in the number of participants who reported adverse events.

In the analysis of differences between and within groups, P values were adjusted for the number of parameters analyzed using Bonferroni correction. SAS statistical package (version 9.1) was used for all analyses.

## Results

### Study Population

A total of 157 participants underwent randomization (79 were assigned to PS-DHA treatment and 78 to placebo treatment). One hundred and thirty one participants completed the double-blind study (66 in the treatment group and 65 in the placebo group). Drop-outs were distributed equally over the two arms and reasons for discontinuation were generally similar across the treatment groups (Table 1). Average age of participants who completed the double-blind study (± SD) was 72.42 ± 8.02 in the PS-DHA group and 72.73 ± 8.25 in the placebo group. There were no significant differences between treatment groups in the incidence of existing disorders including cardiovascular disease and endocrine or metabolic disorders.

**Table 1:** Reasons for study discontinuation

Reason	PS-DHA	Placebo
Protocol violation	2	1

Withdrawn consent	5	6

Adverse events*	5	5

Severe adverse events**	1^#^	1^##^

Sum	13	13

* Adverse events are specified in Table 6

** Classified by the study physician as not related to the study treatment

^# ^Hospitalization due to hyponatremia

^## ^Hospitalization due to atrial fibrillation and epigastric pain

Sixty one participants from the PS-DHA group (PS-DHA continuers) and 61 from the placebo group (PS-naive) continued into the open-label 15 weeks extension period. One participant from the PS-DHA naive group dropped out from the study due to protocol violation. Average age of participants who completed the open-label extension (± SD) was 72.36 ± 7.93 in the PS-DHA continuers group and 72.73 ± 8.31 in the PS-DHA naive group.

### Safety parameters

Physical parameters values during the double-blind phase are presented in Table 2. No significant changes from baseline in resting systolic and diastolic pressure, resting pulse rate and weight were found between treatment groups. A statistically significant increase in weight (0.45 ± 0.04 kg) was detected in the PS-DHA group during the course of the double-blind phase. However, this elevation did not survive Bonferroni correction. Physical parameter values of PS-DHA continuers, who completed the open-label phase, are presented in Table 3. A statistically significant decrease in resting diastolic BP (3.1 ± 0.3 mmHg) and a slight increase in weight (0.61 ± 0.05 kg) were observed following 30 weeks of PS-DHA administration.

**Table 2:** Physical parameters values at baseline and following 15 weeks of double-blind treatment

	PS-DHA			Placebo			
**Variable**	Baseline (mean ± SD)	15 weeks (mean ± SD)	P value^1 ^(within group)	Baseline (mean ± SD)	15 weeks (mean ± SD)	P value^1 ^(within group)	P value^2 ^(between groups)
**Resting systolic BP **(mm Hg)	127.1 ± 13.4	128.4 ± 15.4	0.511	127.6 ± 15.8	127.8 ± 16.5	0.909	0.685

Male	125.4 ± 13.5	126.8 ± 14.6	0.612	129.0 ± 13.0	131.7 ± 12.6	0.244	0.691

Female	128.8 ± 13.3	130.0 ± 16.2	0.676	126.1 ± 18.7	123.3 ± 19.5	0.330	0.331

**Resting diastolic BP **(mm Hg)	73.8 ± 9.2	72.6 ± 9.1	0.299	75.0 ± 7.9	73.3 ± 9.4	0.159	0.746

Male	73.9 ± 9.3	71.7 ± 9.0	0.188	75.4 ± 8.0	73.9 ± 9.7	0.289	0.795

Female	73.6 ± 9.2	73.4 ± 9.3	0.893	74.6 ± 7.8	72.7 ± 9.2	0.357	0.516

**Resting pulse **(beats/minute)	69.4 ± 10.1	69.9 ± 9.5	0.657	66.5 ± 8.4	67.6 ± 10.1	0.394	0.725

Male	69.1 ± 11.8	69.8 ± 10.8	0.648	63.9 ± 7.8	66.1 ± 10.1	0.139	0.482

Female	69.7 ± 8.4	69.9 ± 8.2	0.872	69.6 ± 8.2	69.3 ± 9.6	0.865	0.812

**Weight **(kg)	70.3 ± 11.2	70.8 ± 11.6	0.033*	73.0 ± 12.9	73.3 ± 13.3	0.209	0.622

Male	77.4 ± 9.6	78.0 ± 9.9	0.036*	79.1 ± 11.1	79.5 ± 11.5	0.235	0.664

Female	63.7 ± 8.3	64.0 ± 8.7	0.359	66.1 ± 11.4	66.3 ± 11.9	0.640	0.746

^1 ^Based on two-sided t test for dependent samples. ^2 ^Based on two-sided t test for independent samples

* Statistical significance was not maintained following Bonferonni correction

In the treatment arm, 32 males and 33 females had blood pressure and pulse measurements at baseline and endpoint and 31 males and 33 females had weight measurements at baseline and endpoint. In the placebo arm, 34 males and 29 females had blood pressure and pulse measurements at baseline and endpoint and 33 males and 29 females had weight measurements at baseline and endpoint.

**Table 3:** Physical parameters values of PS-DHA continuers* at baseline and following 30 weeks of treatment

Variable	Baseline (mean ± SD)	30 weeks (mean ± SD)	P value
**Resting systolic BP **(mm Hg)	127.2 ± 13.5	126.3 ± 14.0	0.594

Male	125.7 ± 13.7	126.5 ± 14.3	0.716

Female	128.6 ± 13.4	126.0 ± 14.0	0.366

**Resting diastolic BP **(mm Hg)	73.8 ± 9.4	70.7 ± 9.8	0.006

Male	73.8 ± 9.7	71.6 ± 8.7	0.195

Female	73.8 ± 9.3	69.9 ± 10.7	0.010

**Resting pulse **(beats/minute)	69.4 ± 10.0	70.6 ± 11.9	0.368

Male	69.3 ± 11.6	71.1 ± 13.4	0.412

Female	69.5 ± 8.5	70.1 ± 10.6	0.687

**Weight **(kg)	70.0 ± 11.2	70.7 ± 11.4	0.015

Male	77.1 ± 9.6	77.7 ± 9.8	0.067

Female	63.7 ± 8.5	64.3 ± 8.8	0.104

* include 29 males and 32 females

P value based on two-sided t test for dependent samples

Among PS-DHA naive participants, who received PS-DHA for 15 weeks, there were no significant changes in any of the tested physical parameters at the end of the open-label phase (data not shown).

Biochemical parameters values during the double-blind phase are presented in Table 4. No significant differences between groups were observed in the biochemical parameters, except for minor differences in sodium, calcium, chloride and females triglyceride levels. However, these differences did not survive Bonferroni correction and hence were rendered insignificant. In addition, during the course of the double-blind phase there were few parameters that showed a statistically significant change from baseline in the PS-DHA group, and other parameters in the placebo group. However again, the correction rendered them insignificant.

**Table 4:** Biochemical parameters values at baseline and following 15 weeks of double-blind treatment

	PS-DHA			Placebo			
**Variable**	Baseline (mean ± SD)	15 weeks (mean ± SD)	P value^1 ^(within group)	Baseline (mean ± SD)	15 weeks (mean ± SD)	P value^1 ^(within group)	P value^2 ^(between groups)

**Glucose **(mg/dL)	106.6 ± 34.0	105.9 ± 37.1	0.885	103.4 ± 30.7	111.1 ± 47.3	0.082	0.175

Male	115.1 ± 42.0	110.4 ± 47.4	0.551	105.8 ± 33.6	118.3 ± 59.9	0.076	0.101

Female	98.0 ± 20.7	101.5 ± 22.5	0.330	100.6 ± 27.3	102.9 ± 25.6	0.662	0.847

**Sodium **(mmol/L)	140.6 ± 2.6	140.9 ± 2.7	0.316	140.9 ± 2.1	140.3 ± 2.3	0.014*	0.017*

Male	140.6 ± 2.6	140.9 ± 2.7	0.352	141.2 ± 2.0	140.6 ± 2.1	0.092	0.061

Female	140.5 ± 2.7	140.8 ± 2.7	0.582	140.6 ± 2.2	140.0 ± 2.5	0.079	0.136

**Calcium **(mg/dL)	9.6 ± 0.6	9.6 ± 0.5	0.452	9.4 ± 0.6	9.6 ± 0.5	0.012*	0.038*

Male	9.5 ± 0.4	9.5 ± 0.5	0.966	9.4 ± 0.3	9.5 ± 0.4	0.189	0.413

Female	9.8 ± 0.7	9.6 ± 0.5	0.371	9.5 ± 0.8	9.7 ± 0.5	0.032*	0.051

**Phosphorus **(mg/dL)	3.4 ± 0.6	3.3 ± 0.5	0.103	3.4 ± 0.5	3.3 ± 0.5	0.346	0.807

Male	3.2 ± 0.6	3.1 ± 0.5	0.190	3.2 ± 0.5	3.2 ± 0.5	0.636	0.671

Female	3.7 ± 0.5	3.6 ± 0.5	0.326	3.5 ± 0.6	3.4 ± 0.5	0.397	0.932

**Chloride **(mmol/L)	101.6 ± 3.1	102.4 ± 3.0	0.010*	101.9 ± 3.1	101.8 ± 2.3	0.880	0.047*

Male	101.9 ± 2.8	102.6 ± 3.0	0.094	102.1 ± 3.0	102.1 ± 2.2	1.000	0.262

Female	101.2 ± 3.4	102.2 ± 2.9	0.053	101.6 ± 3.4	101.5 ± 2.4	0.819	0.104

**Potassium **(mmol/L)	4.6 ± 0.5	4.5 ± 0.3	0.031*	4.6 ± 0.5	4.7 ± 0.7	0.703	0.128

Male	4.8 ± 0.7	4.6 ± 0.4	0.095	4.7 ± 0.4	4.7 ± 0.6	0.977	0.221

Female	4.5 ± 0.3	4.4 ± 0.3	0.173	4.6 ± 0.6	4.6 ± 0.8	0.638	0.345

**BUN **(mg/dL)	18.5 ± 4.9	18.9 ± 4.8	0.321	18.6 ± 5.2	19.7 ± 6.7	0.039*	0.262

Male	19.6 ± 4.8	19.9 ± 4.9	0.679	20.5 ± 5.3	21.7 ± 7.2	0.101	0.249

Female**	17.4 ± 4.9	18.0 ± 4.7	0.345	16.3 ± 4.1	17.3 ± 5.3	0.225	0.680

**Creatinine **(mg/dL)	0.9 ± 0.2	0.9 ± 0.2	0.223	0.9 ± 0.2	0.9 ± 0.2	0.416	0.953

Male	1.0 ± 0.2	1.0 ± 0.2	0.507	1.0 ± 0.3	1.0 ± 0.3	0.617	0.420

Female	0.8 ± 0.1	0.8 ± 0.2	0.237	0.7 ± 0.2	0.8 ± 0.2	0.041*	0.265

**Alkaline Phosphatase **(U/L)	68.3 ± 22.5	67.3 ± 22.6	0.331	69.9 ± 30.0	69.4 ± 27.8	0.578	0.726

Male	69.6 ± 24.9	68.0 ± 25.7	0.380	73.3 ± 36.2	72.1 ± 33.0	0.286	0.842

Female	67.1 ± 20.1	66.7 ± 19.4	0.693	66.0 ± 20.8	66.3 ± 20.3	0.862	0.724

**ALT/SGPT **(U/L)	23.9 ± 26.4	20.7 ± 8.4	0.282	19.3 ± 6.4	19.5 ± 5.8	0.733	0.262

Male	28.9 ± 36.3	22.3 ± 9.7	0.273	19.2 ± 5.9	19.2 ± 5.8	1.000	0.276

Female	18.9 ± 6.8	19.0 ± 6.6	0.869	19.5 ± 7.1	19.9 ± 5.8	0.655	0.794

**AST/SGOT **(U/L)	25.2 ± 20.8	22.4 ± 6.1	0.250	21.9 ± 6.2	21.8 ± 5.3	0.879	0.280

Male	28.6 ± 28.6	23.3 ± 7.1	0.276	21.8 ± 5.7	21.3 ± 5.5	0.536	0.329

Female	21.9 ± 6.5	21.5 ± 5.0	0.685	21.9 ± 6.8	22.3 ± 5.0	0.704	0.577

**Total Bilirubin **(mg/dL)	0.6 ± 0.2	0.6 ± 0.3	0.361	0.6 ± 0.3	0.6 ± 0.3	0.673	0.747

Male	0.6 ± 0.3	0.6 ± 0.3	0.097	0.7 ± 0.3	0.7 ± 0.3	0.325	0.753

Female	0.5 ± 0.2	0.5 ± 0.2	0.699	0.5 ± 0.2	0.5 ± 0.2	0.423	0.826

**Total Protein **(g/dL)	7.3 ± 0.4	7.3 ± 0.4	0.190	7.3 ± 0.5	7.4 ± 0.5	0.188	0.936

Male	7.3 ± 0.4	7.3 ± 0.5	0.358	7.3 ± 0.4	7.3 ± 0.5	0.990	0.489

Female	7.3 ± 0.4	7.3 ± 0.4	0.362	7.4 ± 0.6	7.5 ± 0.4	0.091	0.443

**Triglycerides **(mg/dL)	141.6 ± 61.4	133.0 ± 70.0	0.133	128.9 ± 54.8	131.6 ± 58.5	0.677	0.186

Male	138.2 ± 68.1	133.8 ± 79.6	0.577	131.6 ± 54.9	121.7 ± 42.2	0.198	0.611

Female	145.1 ± 54.8	132.2 ± 60.0	0.132	125.9 ± 55.4	142.8 ± 71.9	0.103	0.025*

**HDL **(mg/dL)	54.8 ± 12.4	56.4 ± 13.4	0.118	53.7 ± 14.1	54.4 ± 14.1	0.430	0.505

Male	48.8 ± 9.6	50.4 ± 10.5	0.044*	48.3 ± 10.2	49.2 ± 11.2	0.348	0.604

Female	60.9 ± 12.1	62.4 ± 13.4	0.404	59.9 ± 15.4	60.3 ± 14.9	0.785	0.635

**LDL **(mg/dL)	97.6 ± 31.4	98.6 ± 31.7	0.745	98.9 ± 30.9	99.1 ± 25.6	0.940	0.853

Male	88.6 ± 30.4	91.0 ± 31.8	0.287	94.4 ± 28.9	92.9 ± 21.2	0.656	0.332

Female	106.6 ± 30.2	106.2 ± 30.2	0.946	104.1 ± 32.8	106.2 ± 28.5	0.672	0.743

**Total cholesterol **(mg/dL)	177.5 ± 38.2	181.1 ± 35.2	0.402	178.0 ± 37.2	179.4 ± 33.7	0.650	0.687

Male	164.6 ± 31.2	167.6 ± 33.1	0.283	168.5 ± 33.2	166.0 ± 26.3	0.487	0.226

Female	190.3 ± 40.6	194.5 ± 32.4	0.613	188.7 ± 39.0	194.6 ± 35.1	0.284	0.856

^1 ^Based on two-sided t test for dependent samples. ^2 ^Based on two-sided t test for independent samples

* Statistical significance was not maintained following Bonferonni correction

In the treatment arm, 33 males and 33 females had biochemical blood measurements at baseline and endpoint.

In the placebo arm, 34 males and 30 females had biochemical blood measurements at baseline and endpoint.

** In the placebo arm 29 females had BUN measurements at baseline and endpoint.

Hematological parameters values during the double-blind phase are presented in Table 5. No significant differences between groups were observed, except for a minor difference in the neutrophils count of the female population. Again, this difference did not survive Bonferroni correction and hence was rendered insignificant. In addition, during the course of the double-blind phase there were few hematological parameters that showed slight changes from baseline in the PS-DHA group, and other parameters in the placebo group, however again, the correction rendered them insignificant.

**Table 5:** Hematological parameters values at baseline and following 15 weeks of double-blind treatment

	PS-DHA			Placebo			
**Variable**	Baseline (mean ± SD)	15 weeks (mean ± SD)	P value^1 ^(within group)	Baseline (mean ± SD)	15 weeks (mean ± SD)	P value^1 ^(within group)	P value^2 ^(between groups)

**White blood cell count **(×10^3^/cmm)	6.8 ± 1.8	6.5 ± 1.6	0.083	7.1 ± 2.0	6.7 ± 1.9	0.011*	0.434

Male	6.8 ± 1.7	6.4 ± 1.5	0.020*	7.3 ± 2.3	7.1 ± 2.2	0.298	0.467

Female	6.7 ± 1.9	6.7 ± 1.6	0.829	6.9 ± 1.4	6.3 ± 1.3	0.009*	0.066

**Red blood cell count **(×10^6^/cmm)	4.6 ± 0.5	4.6 ± 0.5	0.441	4.6 ± 0.4	4.6 ± 0.4	0.332	0.765

Male	4.7 ± 0.5	4.7 ± 0.5	0.544	4.6 ± 0.4	4.6 ± 0.4	0.491	0.925

Female	4.5 ± 0.4	4.4 ± 0.4	0.622	4.5 ± 0.4	4.5 ± 0.4	0.488	0.760

**Hematocrit **(%)	41.2 ± 4.2	40.7 ± 4.3	0.113	41.6 ± 3.6	40.9 ± 3.6	0.045*	0.504

Male	42.5 ± 4.3	42.1 ± 4.4	0.229	42.6 ± 3.6	42.0 ± 3.4	0.100	0.763

Female	39.8 ± 3.7	39.4 ± 3.8	0.309	40.5 ± 3.2	39.6 ± 3.5	0.193	0.548

**Hemoglobin **(g/dL)	13.7 ± 1.5	13.7 ± 1.5	0.434	13.7 ± 1.2	13.6 ± 1.2	0.213	0.669

Male	14.3 ± 1.4	14.3 ± 1.3	0.706	14.1 ± 1.2	13.9 ± 1.2	0.181	0.623

Female	13.1 ± 1.4	13.0 ± 1.4	0.481	13.3 ± 1.1	13.2 ± 1.1	0.494	0.856

**Platelet count **(×10^3^/cmm)	221.5 ± 62.7	226.8 ± 60.5	0.374	226.9 ± 73.7	230.8 ± 68.8	0.347	0.839

Male	200.1 ± 44.5	204.0 ± 44.3	0.479	202.8 ± 58.3	210.0 ± 61.1	0.128	0.648

Female	243.5 ± 71.3	250.2 ± 66.4	0.536	255.4 ± 80.6	255.3 ± 70.4	0.980	0.592

**Netrophils **(%)	62.8 ± 6.5	62.4 ± 6.4	0.514	63.8 ± 8.7	62.9 ± 8.6	0.207	0.543

Male	65.1 ± 6.0	64.4 ± 5.7	0.390	64.8 ± 9.0	64.8 ± 9.2	0.968	0.569

Female	60.4 ± 6.2	60.5 ± 6.7	0.969	62.6 ± 8.4	60.7 ± 7.5	0.097	0.147

**Netrophils Absolute **(×10^9^/L)	4.3 ± 1.3	4.1 ± 1.0	0.080	4.5 ± 1.4	4.2 ± 1.4	0.037*	0.579

Male	4.5 ± 1.3	4.1 ± 1.1	0.027*	4.7 ± 1.5	4.6 ± 1.7	0.675	0.265

Female	4.1 ± 1.2	4.0 ± 1.0	0.814	4.4 ± 1.2	3.8 ± 1.0	0.010*	0.046*

**Lymphocytes **(%)	26.0 ± 6.1	26.1 ± 5.8	0.887	25.8 ± 8.7	26.4 ± 8.1	0.324	0.478

Male	23.8 ± 5.6	24.4 ± 5.1	0.453	24.5 ± 8.8	24.4 ± 8.7	0.893	0.529

Female	28.2 ± 5.8	27.8 ± 6.0	0.447	27.3 ± 8.4	28.8 ± 6.9	0.171	0.116

**Lymphocytes Absolute **(×10^9^/L)	1.8 ± 0.6	1.7 ± 0.5	0.167	1.9 ± 1.1	1.8 ± 0.8	0.051	0.449

Male	1.6 ± 0.5	1.6 ± 0.4	0.234	1.9 ± 1.4	1.7 ± 1.0	0.115	0.468

Female	1.9 ± 0.6	1.8 ± 0.6	0.458	1.8 ± 0.6	1.8 ± 0.5	0.238	0.819

**Monocytes **(%)	5.8 ± 1.4	6.0 ± 1.5	0.119	5.6 ± 1.0	5.9 ± 1.4	0.093	0.918

Male	5.9 ± 1.5	6.1 ± 1.4	0.343	5.7 ± 0.9	5.9 ± 1.3	0.299	0.976

Female	5.6 ± 1.4	5.9 ± 1.6	0.220	5.5 ± 1.1	5.8 ± 1.5	0.193	0.895

**Monocytes Absolute **(×10^9^/L)	0.4 ± 0.1	0.4 ± 0.1	0.731	0.4 ± 0.1	0.4 ± 0.1	0.565	0.516

Male	0.4 ± 0.1	0.4 ± 0.1	0.497	0.4 ± 0.1	0.4 ± 0.1	0.890	0.556

Female	0.4 ± 0.1	0.4 ± 0.1	0.284	0.4 ± 0.1	0.4 ± 0.1	0.359	0.159

**Eosinophils **(%)	3.0 ± 2.5	3.1 ± 3.3	0.365	2.5 ± 1.6	2.5 ± 1.5	0.844	0.540

Male	2.7 ± 1.5	2.8 ± 2.0	0.535	2.8 ± 1.8	2.7 ± 1.7	0.832	0.533

Female	3.3 ± 3.3	3.5 ± 4.2	0.513	2.2 ± 1.4	2.3 ± 1.2	0.660	0.796

**Eosinophils Absolute **(×10^9^/L)	0.2 ± 0.3	0.2 ± 0.3	0.853	0.2 ± 0.1	0.2 ± 0.1	0.227	0.400

Male	0.2 ± 0.2	0.2 ± 0.2	0.421	0.2 ± 0.1	0.2 ± 0.1	0.280	0.943

Female	0.2 ± 0.4	0.3 ± 0.5	0.382	0.1 ± 0.1	0.1 ± 0.1	0.513	0.284

**Basophils **(%)	0.7 ± 0.3	0.6 ± 0.3	0.041*	0.7 ± 0.3	0.6 ± 0.3	0.101	0.684

Male	0.7 ± 0.3	0.6 ± 0.3	0.207	0.6 ± 0.3	0.6 ± 0.3	0.099	0.882

Female	0.8 ± 0.2	0.6 ± 0.3	0.113	0.7 ± 0.4	0.7 ± 0.3	0.515	0.482

**Basophils Absolute **(×10^9^/L)	0.1 ± 0.0	0.0 ± 0.0	0.006*	0.1 ± 0.0	0.0 ± 0.0	0.019*	0.854

Male	0.1 ± 0.0	0.0 ± 0.0	0.035*	0.1 ± 0.0	0.0 ± 0.0	0.058	0.928

Female	0.1 ± 0.0	0.0 ± 0.0	0.077	0.1 ± 0.0	0.0 ± 0.0	0.174	0.701

**Large Unstained Cells **(%)	1.7 ± 0.6	1.7 ± 0.5	1.000	1.6 ± 0.6	1.7 ± 0.6	0.162	0.306

Male	1.8 ± 0.6	1.8 ± 0.5	0.616	1.6 ± 0.6	1.6 ± 0.6	0.623	0.483

Female	1.7 ± 0.7	1.7. ± 0.6	0.701	1.7 ± 0.5	1.9 ± 0.6	0.139	0.434

**Large Unstained Cells Absolute **(×10^9^/L)	0.1 ± 0.0	0.1 ± 0.0	0.652	0.1 ± 0.1	0.1 ± 0.1	0.571	0.948

Male	0.1 ± 0.0	0.1 ± 0.0	0.243	0.1 ± 0.1	0.1 ± 0.1	0.568	0.753

Female	0.1 ± 0.1	0.1 ± 0.0	0.784	0.1 ± 0.0	0.1 ± 0.0	0.847	0.742

**MCV **(μ^3^)	90.1 ± 4.1	89.6 ± 4.4	0.133	91.0 ± 4.3	90.1 ± 4.3	0.049*	0.506

Male	90.7 ± 4.4	90.2 ± 4.4	0.204	92.2 ± 4.3	91.4 ± 4.6	0.211	0.780

Female	89.5 ± 3.9	89.1 ± 4.31	0.397	89.6 ± 3.9	88.7 ± 3.6	0.124	0.503

**MCH **(pg)	30.1 ± 2.1	30.1 ± 1.9	0.850	30.0 ± 1.5	30.0 ± 1.4	0.940	0.861

Male	30.6 ± 2.2	30.7 ± 2.0	0.651	30.4 ± 1.6	30.4 ± 1.5	0.842	0.658

Female	29.5 ± 1.8	29.5 ± 1.8	0.801	29.5 ± 1.1	29.5 ± 1.1	0.910	0.810

**MCHC **(%)	33.4 ± 1.6	33.6 ± 1.4	0.108	33.0 ± 1.2	33.3 ± 0.9	0.041*	0.673

Male	33.8 ± 1.9	34.1 ± 1.7	0.116	33.0 ± 1.2	33.2 ± 1.1	0.267	0.794

Female	32.9 ± 1.1	33.1 ± 0.9	0.533	32.9 ± 1.1	33.3 ± 0.7	0.061	0.335

^1 ^Based on two-sided t test for dependent samples. ^2 ^Based on two-sided t test for independent samples

* Statistical significance was not maintained following Bonferonni correction

In the treatment arm, 33 males and 32 females had hematological blood measurements at baseline and endpoint.

In the placebo arm, 33 males and 28 females had hematological blood measurements at baseline and endpoint.

### Adverse Events

Adverse events reported during the course of the double-blind phase are presented in Table 6. Twenty participants from the PS-DHA and 11 participants from the placebo group reported adverse events (29 and 15 adverse events, respectively). There were no significant differences between the study groups in the number of participants who reported an adverse event (P = 0.078) or in the number of reported adverse events (P = 0.087). Ten participants from the PS-DHA group and 8 participants from the placebo group were classified by the study physicians as suffering from related or probably related adverse events (16 and 11 adverse events, respectively). Again, there were no significant differences between the study groups in the number of participants who reported an adverse event (P = 0.637) or in the number of reported adverse events, classified as related or probably related to the study treatment (P = 0.472).

**Table 6:** Adverse events reported during the course of the double-blind phase*

	PS-DHA		Placebo	
**Adverse event**	**Number of related or probably related adverse events**	**Number of not-related or probably not related adverse events**	**Number of related or probably related adverse events**	**Number of not-related or probably not related adverse events**

**Adverse events among study drop-outs**				

Gastrointestinal discomfort	4		1	

Rush	1			

Increased appetite and weight			1	

Strange general feeling			1	

Headache			2	

dizziness			2	

Mood swings			1	

*SUM*	*5 events in 5 participants*		*8 events in 5 participants*	

**Adverse events among study completers**				

Gastrointestinal discomfort	9	3	1	2

Weight loss	1	1		

Gastritis		1		

Headache	1	1		

Pneumonia		2		

Hypertension		2		1

Hypothyroidism		1		

Back pain		1		

Leg wound		1		

Redness in the mouth			1	

Pruritus			1	

Eyes inflammation				1

*SUM*	*11 events in 5 participants*	*13 events in 10 participants*	*3 events in 3 participants*	*4 events in 3 participants*

* Judged by the study physicians as related, probably related, not related or probably not related to the study treatment

During the course of the double-blind phase, there were 5 severe adverse events (suspected acute diverticulitis, benign prostatic hyperplasia surgery, and 3 hospitalizations due to hyponatremia, bradycardia and abdominal discomfort) in 5 participants in the PS-DHA group and 2 severe adverse events (atrial fibrillation and epigastric pain leading to hospitalization) in one participant in the placebo group. All the severe adverse events were classified by the study physicians as not related or probably not related to the study treatment.

Adverse events reported during the course of the open-label extension are presented in Table 7. Ten participants from the PS-DHA continuers reported 12 adverse events of which 3 were classified by the study physicians as related or probably related to the study treatment. Six participants from the PS-DHA naive group reported 9 adverse events; none of which were classified as related or probably related to the study treatment.

**Table 7:** Adverse events reported during the course of the open-label extension*

PS-DHA continuers		PS-DHA naive**	
**Adverse event**	**Number of related or probably related adverse events**	**Number of not-related or probably not related adverse events**	**Number of not-related or probably not related adverse events**

Gastrointestinal discomfort	1		3

Hypertension		1	3

Hypotension		1	

Fall		1	

Tenesmus	1		

Amaurosis fugax		1	

Mild bleeding due to Hemorrhoids		1	

Platelets decrease		1	

Hand Fracture		1	

Hyperlipidemia			1

Dizziness		1	

Urinal tract infection			1

Arm pigmentation			1

Headache	1	1	

**SUM**	**3 events in 3 participants**	**9 events in 7 participants**	**9 events in 6 participants**

* Judged by the study physicians as related, probably related, not related or probably not related to the study treatment

** There were no adverse events classified by the study physicians as related or probably related to the study treatment

During the course of this phase, there were 4 severe adverse events (elective hospitalization for computed tomography urography, and chest pains, ear cholesteotoma and prostate surgery, leading to hospitalization), all classified by the study physicians as not related or probably not related to the study treatment.

## Discussion

PS is widely used as an over the counter (OTC) preparation in the aim of improving general health and particularly cognitive functions of elderly people. Traditionally, PS has been extracted from bovine brain; however, recently, the BSE epidemic necessitated the finding of alternative safe sources such as SB-PS. SB-PS, however, differs considerably from BC-PS mainly in the absence of DHA which is the predominant omega-3 LC-PUFA in the mammalian central nervous system. Observational and epidemiological studies have associated omega-3 LC-PUFA consumption with a reduced risk of impaired cognitive function in middle-aged population [19], and with a reduced risk of dementia [20,21]. This association has been supported by interventional studies [22-24].

To gain the benefits of mammalian PS without the attendant risks, a safe-sourced PS with omega-3 LC-PUFA attached to its backbone (PS- omega-3) was developed. This compound was recently found to improve the symptoms of children with impaired visual sustained attention [15] and to protect middle-aged rats from scopolamine-induced deleterious effects [16]. The mechanism by which PS- omega-3 exerts its effects is not fully understood, however PS has been found to regulate key proteins in neuronal membranes, including sodium/calcium ATPase [25], protein kinase C [26] and Raf-1 protein kinase [27]. PS was also found to influence neurotransmitter activity, such as the release of acetylcholine, dopamine and noradrenaline [28]. In addition, PS- omega-3 was found to significantly increase DHA level in brains of middle-aged rats [16].

We have recently reported that a novel PS preparation (PS-DHA) may improve cognitive performance in non-demented elderly with memory complaints [17] and the safety results of this study are described herein.

Participants who discontinued the double-blind phase due to adverse events were distributed equally over the two study arms. No significant findings were observed in the tested physical, hematological or biochemical parameters following 15 weeks of treatment and there was no significant difference in the frequency of adverse events between the groups.

No subject discontinued the open-label phase due to an adverse event and there were no significant findings in the tested physical parameters.

Overall, the treatment was well tolerated with the most frequent symptom related to gastrointestinal discomfort. These symptoms were considered mild by the subjects and the physicians, and were previously reported for other PS compounds [29]. No severe adverse events were classified by the study physicians as related or probably related to the study treatment.

## Conclusions

Taken in combination with the positive cognitive effects of the PS-DHA preparation reported previously [17], the study results support the safe use of PS-DHA for elderly people with memory complaints at a dosage of 300 mg PS/day for 15 weeks, or at a lower dose (100 mg PS/day) for 30 weeks.

## Competing interests

This study was funded by Enzymotec LTD, Israel. YR, TC and YH are employees of Enzymotec Ltd, and ADK served as consultant to the study.

## Authors' contributions

ADK, YR and YH interpreted the data and wrote the draft of this manuscript. The study protocol was designed by VV, ADK, YR and TC. VV and ADK were responsible for patient recruitment and management. All authors were involved in revising the manuscript and gave final approval for publication.

## Pre-publication history

The pre-publication history for this paper can be accessed here:

http://www.biomedcentral.com/1471-2377/11/79/prepub
